# Estimation of changes in the force of infection for intestinal and urogenital schistosomiasis in countries with schistosomiasis control initiative-assisted programmes

**DOI:** 10.1186/s13071-015-1138-1

**Published:** 2015-10-24

**Authors:** Michael D. French, Thomas S. Churcher, Joanne P. Webster, Fiona M. Fleming, Alan Fenwick, Narcis B. Kabatereine, Moussa Sacko, Amadou Garba, Seydou Toure, Ursuline Nyandindi, James Mwansa, Lynsey Blair, Elisa Bosqué-Oliva, Maria-Gloria Basáñez

**Affiliations:** Schistosomiasis Control Initiative, Faculty of Medicine, Imperial College London, St. Mary’s Hospital, Norfolk Place, London, W2 1PG UK; Department of Infectious Disease Epidemiology, School of Public Health, Faculty of Medicine, Imperial College London, Norfolk Place, London, W2 1PG UK; Vector Control Division, Ministry of Health, Kampala, Uganda; Ministère de la Santé, Bamako, Mali; Ministère de la Santé Publique (now WHO), Niamey, Niger; Ministry of Health, Ougadougou, Burkina Faso; Ministry of Health and Social Welfare, Dar es Salaam, Tanzania; Department of Pathology and Microbiology, University of Zambia School of Medicine, University Teaching Hospital, Lusaka, Zambia; Present address: World Health Organization, 20, avenue Appia, 1211 Geneva 27, Switzerland; Present address: The END FUND, New York, NY USA; Present address: Department of Pathology and Pathogen Biology, Centre for Emerging, Endemic and Exotic Diseases (CEEED), Royal Veterinary College, University of London, Hawkshead Campus, Herts, AL97TA London, UK

**Keywords:** Schistosomiasis, Mass drug administration, Praziquantel, *Schistosoma mansoni*, *Schistosoma haematobium*, Force of infection, sub-Saharan Africa, Mathematical modelling

## Abstract

**Background:**

The last decade has seen an expansion of national schistosomiasis control programmes in Africa based on large-scale preventative chemotherapy. In many areas this has resulted in considerable reductions in infection and morbidity levels in treated individuals. In this paper, we quantify changes in the force of infection (*FOI*), defined here as the per (human) host parasite establishment rate, to ascertain the impact on transmission of some of these programmes under the umbrella of the Schistosomiasis Control Initiative (SCI).

**Methods:**

A previous model for the transmission dynamics of *Schistosoma mansoni* was adapted here to *S. haematobium*. These models were fitted to longitudinal cohort (infection intensity) monitoring and evaluation data. Changes in the *FOI* following up to three annual rounds of praziquantel were estimated for Burkina Faso, Mali, Niger, Tanzania, Uganda, and Zambia in sub-Saharan Africa (SSA) according to country, baseline endemicity and schistosome species. Since schistosomiasis transmission is known to be highly focal, changes in the *FOI* at a finer geographical scale (that of sentinel site) were also estimated for *S. mansoni* in Uganda.

**Results:**

Substantial and statistically significant reductions in the *FOI* relative to baseline were recorded in the majority of, but not all, combinations of country, parasite species, and endemicity areas. At the finer geographical scale assessed within Uganda, marked heterogeneity in the magnitude and direction of the relative changes in *FOI* was observed that would not have been appreciated by a coarser-scale analysis.

**Conclusions:**

Reductions in the rate at which humans acquire schistosomes have been achieved in many areas of SSA countries assisted by the SCI, while challenges in effectively reducing transmission persist in others. Understanding the underlying heterogeneity in the impact and performance of the control intervention at the level of the transmission site will become increasingly important for programmes transitioning from morbidity reduction to elimination of infection. Such analyses will require a fine-scale approach. The lack of association found between programmatic variables, such as therapeutic treatment coverage (recorded at district level) and changes in *FOI* (at sentinel site level) is discussed and recommendations are made.

## Background

Recent years have seen a great expansion of national schistosomiasis control programmes, mostly based on large-scale preventive chemotherapy—also known as mass drug administration (MDA)—with praziquantel, resulting in significant successes in controlling infection prevalence, intensity, and morbidity [1, 2]. What is not yet clear is the benefit of such large-scale treatment to the wider community via reductions in transmission. These reductions will be manifested via a decrease in the force of infection (*FOI*) which, for macroparasitic infections, is defined as the *per capita* rate at which a host acquires new infections [3]. The precise understanding of the *FOI* can be interpreted in a number of different ways according to which stage of the parasites’ life-cycle is of interest. The number of adult schistosomes within a host can rarely be measured directly, so parasitological surveys typically rely on faecal egg counts as a proxy for parasite intensity. Therefore, routinely used diagnostic tools cannot identify newly established parasites until they reach patency and reproduce successfully.

For the purposes of this paper, the *FOI* is defined as the rate at which new incoming worms establish into adult parasites and reach patency (initiate detectable egg production) in the human host population, and is conceptually similar to the use of age-specific rates of reinfection after treatment to measure the force of infection used by other researchers [4]. However, this may differ from the rate at which the host population is infected by other parasitic stages, such as by cercariae from the environment, which has been the approach used historically via the use of snail studies or cercariometry [5–7].

Since schistosomiasis transmission models began to be developed in earnest in the 1960s, significant advances were the formulation of the reproduction number (in prevalence models) to estimate rates of infection and clearance [8, 9]; inclusion of mating probabilities and transmission breakpoints (in intensity frameworks) to examine how these influence the establishment, persistence, and elimination of the infection [10–12]; incorporation of parasite latency and snail mortality [3, 12–14]; and the comparison of model outputs to field data to investigate heterogeneity in infection [15], among others. Although some authors have favoured the use of prevalence-based models [16], the use of (deterministic) immigration-death, intensity frameworks was adopted in [17], where such approaches describe the rate of change of the number of schistosomes per person with respect to time and host age. These were used to estimate the rate of parasite acquisition (*FOI*), with this rate found to be reliant on the intensity of incoming worms and the load of already established parasites. Although such models track the number of adult worms in human hosts, they either calculate the prevalence of patent infections in snails [18, 19], or assume that the schistosome population in snails and the parasite larval population (miracidia; cercariae) in the environment are at equilibrium given the disparity in the respective life-spans [3]. The latter was the approach adopted by the authors of the deterministic model EpiSchisto®[20], upon which the code used in this study is based.

In a previous paper we used this approach to estimate changes in the *FOI* relative to that at baseline, for cohorts of individuals treated annually for three years with praziquantel against intestinal schistosomiasis in Uganda [21]. The extensive datasets of the Schistosomiasis Control Initiative (SCI), allowed regions within Uganda to be classified according to underlying levels of average infection intensity at baseline measured in a cohort of school-aged children and adults, namely, high endemicity: ≥400 eggs per gram of faeces [epg]; moderate endemicity: 100–399 epg, and low endemicity: 1–99 epg [22]. Substantial and statistically significant reductions in the *FOI* were observed following a single treatment round in areas of low intensity of infection (76 % reduction; 95 % confidence interval [95 % CI]: 59–100 %), and following two rounds in those of moderate (66 %; 95 % CI: 26–87 %) and high (63 %; 95 % CI: 37–78 %) intensity. It was concluded that the programme not only benefited those individuals receiving treatment, but also those who missed treatment, due to the ensuing decrease in environmental transmission. Demonstrating quantitatively the impact of anthelmintic interventions is crucial to ensure their sustainability, the engagement of communities and funders, and the ultimate estimation of their cost effectiveness [23].

Notwithstanding the usefulness of examining the impact of interventions at relatively broad epidemiological levels, these are likely to combine areas that differ in terms of their transmission patterns. In reality, schistosomiasis is a focal disease, and considerable heterogeneity can often be observed in infection in neighbouring communities, and even within communities [24]. In this paper the earlier modelling approach is extended to calculate relative changes in the *FOI* according to endemicity level in six countries that received SCI support between 2003 and 2008 for both *Schistosoma mansoni* and *S. haematobium*. The heterogeneity of such results at a smaller, finer geographical scale is also explored, using *S. mansoni* in Uganda as an illustrative example.

## Methods

### Datasets and cohorts

**Fig. 1:**
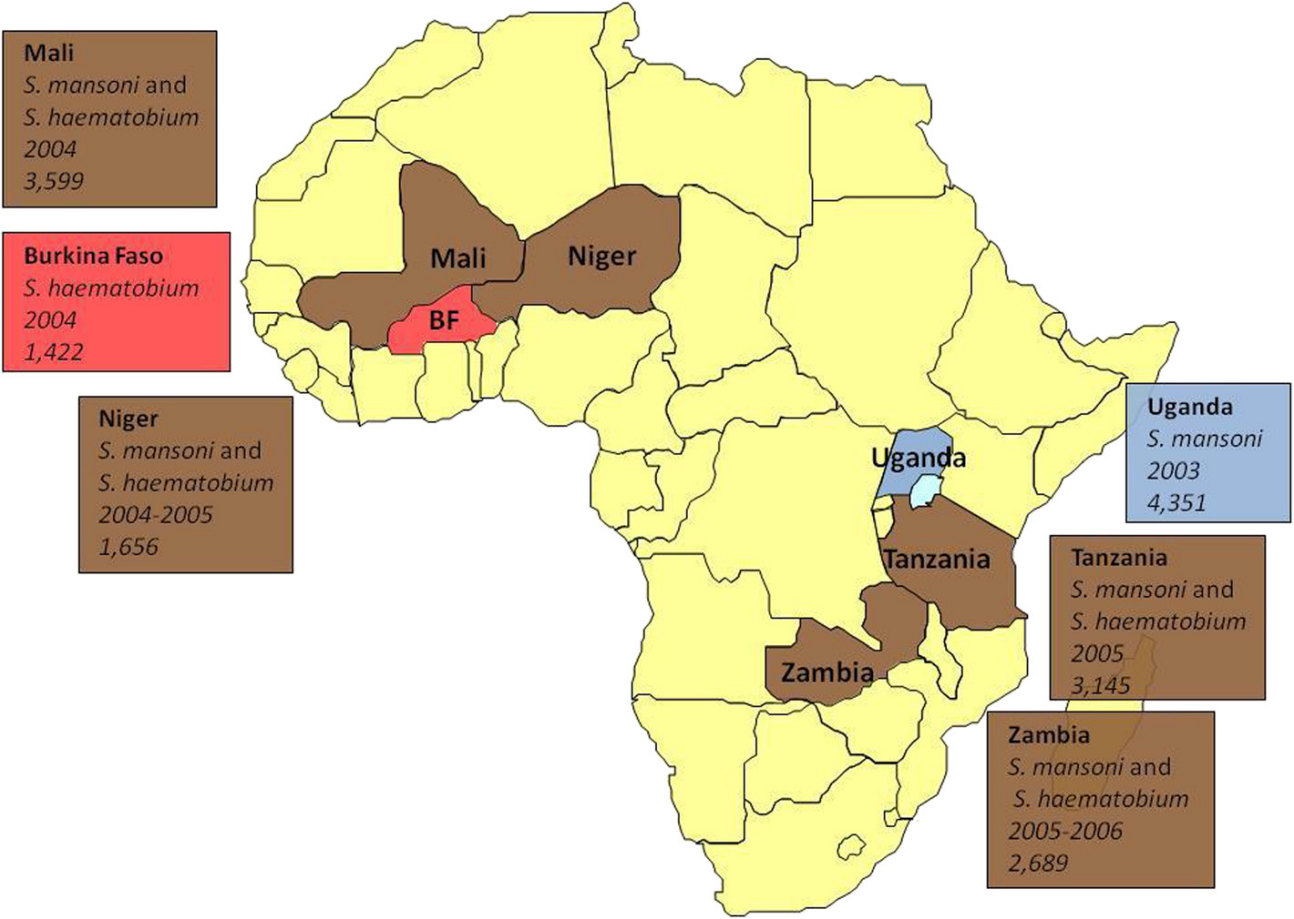
Map of Africa showing: those SCI-assisted countries that provided datasets included in this study, species of schistosome found in each, cohort sizes, and year of commencement of control programme

A full description of the data collection design for the monitoring and evaluation (M&E) components of the SCI treatment programmes is given elsewhere [2]. In brief, data were collected for the six countries that implemented national or near-national treatment for schistosomiasis (and soil-transmitted helminthiasis), soon after the inception of the SCI in 2002. These were three countries from West Africa (Burkina Faso, Mali, Niger), and three from East Africa (Tanzania, Uganda, Zambia), which provided datasets on either *S. mansoni* or *S. haematobium* or both (Table 1 and Fig. 1). The M&E approach was designed using a statistical framework [2] to ensure that were a reduction in infection levels occurring, such a reduction would be identifiable and measurable [25].

**Table 1:** Summary of longitudinal cohorts in three West African and three East African countries selected to receive assistance by the Schistosomiasis Control Initiative for implementation of preventive chemotherapy programmes against schistosomiasis (and soil-transmitted helminthiasis)

Country	Species	Cohort Size at Baseline (No. schools)	BL Year	Tx BL	FY1 Year, Numbers, (Follow-up rate), Number of schools	Tx FY1	FY2 Year, Numbers, (Follow-up rate), Number of schools	Tx FY2	FY3 Year, Numbers, (Follow-up rate), Number of schools	Tx FY3	Age-range of school cohorts at baseline	Proportion of females at BL (%)	Relevant Publications
West Africa													
Burkina Faso	*S. haematobium*	1,422 (16 schools)	2004	Y	2005, 996 (70.0 %) 16 schools	N	2006, 770 (54.1 %) 16 schools	Y	2007, 564 (39.7 %) 15 schools	N	6-12	46.6	[2, 90, 91, 93]
Mali	*S. mansoni & S. haematobium*	3,599 (33 schools)	Mar-Jun 2004	Y	May-Jun 2005 and Apr – May 2006 1995 (55.4 %) 24 schools	Y	Mar-Apr 2006 and May-June 2007 (1511 (42.0 %) 27 schools	Y	ND	Y	7-14	49.9	[2, 26, 90, 91]
Niger	*S. haematobium*	1,656 (8 schools)	Oct-Nov 2004 and Mar-May 2005	Y	Oct-Dec 2005 and Mar-Apr 2006,1440 (87.0 %) 8 schools	Y	Nov-Dec 2006 and Jan-May 2007, 1193 (72.0 %) 8 schools	Y	ND	x	7,8,11	45.5	[2, 82, 90, 91, 94]
East and Southern Africa													
Tanzania	*S. mansoni & S. haematobium*	3,145 (20 schools)	2005	Y	2006, 2235 (71.1 %) 19 schools	Y	-	N	2008, 1076 (34.2 %) 14 schools	N	7-12	56.9	[2, 92]
Uganda	*S. mansoni*	4,351 (37 schools)	2003	Y	2004, 2815 (64.7 %) 37 schools	Y	2005, 1871 (43.0 %) 37 schools	Y	2006, 1156 (26.6 %) 33 schools	Y	6-8, 11	49.1	[1, 2, 92, 95]
Zambia	*S. mansoni & S. haematobium*	2,689 (22 schools)	Jul-Aug 05 and May –Jun 06	Y	Sep 2006 and May-Jun 2007, 1633 (56.9 %) 21 schools	Y	-	N	-	x	7-12	53.4	[2, 92]

*BL* baseline, *Tx* treatment round, *FY1* follow-up year 1, *FY2* follow-up year 2, *FY3* follow-up year 3, *Y* treatment received, *N* treatment not received, *ND* not done

There are two types of cohorts that comprise the SCI data. Longitudinal cohorts consist of school-age children in primary education (6–12 years or 7–12 years depending on age at first enrolment) followed up every year for 1 year (Zambia) or 2–3 years (the remaining countries) (Table 1). Community cohorts focus on collecting data from adults but also include children (age range of 4 – 88 years); these are designed to capture individuals longitudinally but due to the higher drop-out rate are often of a cross-sectional design.

M&E cohorts are often assumed (either explicitly or implicitly) to be representative of the wider community. This may be true at baseline, where extra efforts are often made to attract non-enrolled school-age children. However, at follow-up, when (systematic) non-compliance may play a significant role, those children more heavily infected or too ill, engaged in work, or living in more distant locations may not be able to attend school for re-treatment. To investigate whether the longitudinal cohorts were truly representative of the study populations of interest, the baseline values of infection intensity and prevalence in those children who were followed for each year of the study were compared with those of children who were lost to follow-up [26] via normal distribution z-tests for large samples [27]. The cohort retention rate was calculated as the proportion of individuals recruited into the cohort at baseline who subsequently were positively identified at each of the following treatment rounds. Differences in the cohort retention rate with respect to age were calculated using logistic regression with age as the dependent variable and whether the individual was retained in the cohort as the independent variable.

### Sampling and descriptive statistics

For *S. mansoni*, infection intensity was estimated as the arithmetic mean of either four Kato-Katz slides taken across two consecutive days (two slides on day one and two slides on day two, in Uganda, Tanzania, and Zambia) or of two Kato-Katz slides taken on one day (Mali, Niger, Burkina Faso). Infection intensity is expressed as eggs per gram of faeces (epg) [28]. For *S. haematobium*, infection intensity was estimated from either a single urine filtration sample (Uganda, Tanzania, Zambia, Mali, and Burkina Faso) or a double urine filtration from a single urine sample (Niger). Infection intensity is expressed as eggs per 10 ml urine (e/10 ml) [22].

The relationship between adult worm burden and egg output is poorly understood. This is in part caused by variations in egg output by day, uneven distribution of eggs in the excreta, imperfect sensitivity of diagnostic methods, and the fact that only a proportion of the eggs will exit the body [29–33]. However, attempts to define the relationship between egg output and underlying worm burden have concluded that there may be a positive and linear relationship for both *S. mansoni* [20] and *S. haematobium* [34]. In contrast, analysis of the human autopsy dataset of Cheever [35] suggested some measure of negative density dependence in *S. mansoni* [36]. In the present study, and for parsimony, a density-independent relationship was assumed between female worm burden and egg output, with each *S. mansoni* female producing on average of 5.26 epg [20], and each *S. haematobium* female a mean of 3.60 e/10 ml [34].

Arithmetic means of infection intensity were used as measures of central tendency [37], and their ninety five percent confidence intervals (95 % CI) were calculated using the normal approximation for large sample sizes [27]. Point estimates of prevalence of infection and categories of infection (as described below) were also calculated and the normal approximation to the binomial distribution was used to estimate their 95 % CI given the large sample sizes available [27]. All statistical analyses were carried out in the freely available software R [38].

### Endemicity levels

To follow the same methodology for categorization of endemicity of French et al. [21], the datasets were classified into areas of low, moderate and high underlying endemicity, based on the average infection intensity for each sentinel site (school/community) at baseline, prior to large-scale implementation of SCI-supported praziquantel treatment programmes (although the possibility that there had been previous, smaller-scale ad-hoc treatment programmes cannot be totally discounted). Intestinal schistosomiasis areas were allocated into high (≥400 epg), moderate (100–399 epg) and low (1–99 epg) infection intensity areas as described above. For urogenital schistosomiasis, areas they were classified into high (≥50 e/10 ml) and low (1–49 e/10 ml) intensity areas [22]. In both Mali and Tanzania the numbers of highly-infected *S. mansoni* sentinel sties was low, and so these were combined into a single high/moderate category.

### Mathematical modelling

#### Schistosoma mansoni

Full details of the population dynamics model for *S. mansoni*can be found in French et al. (2010) [21] and in the supplementary information of that paper. These authors modified an earlier framework presented by Chan et al. [20] known as EpiSchisto®. In turn, EpiSchisto® was based on previous work by Anderson and May [3, 17] developing the use of immigration-death models. The rate of change in mean adult *S. mansoni*worm burden (*M*) (after the conversion from egg output to adult female burden given previously of 5.26 epg per mated female worm) with respect to host age (*a*) and time (*t*) can be described by the following immigration-death equation,

1 $$ \frac{\partial M\left(a,t\right)}{\partial t}+\frac{\partial M\left(a,t\right)}{\partial a}=\varLambda (a)-{\mu}_MM\left(a,t\right), $$

where *Λ*(*a*) is the net *FOI* at age *a*, and *μ*_*M*_ is the per worm death rate of established (mature) adult worms. In turn, *Λ*(*a*) is given by Eq. 2,

2 $$ \varLambda (a)={\lambda}_B{\zeta}_P\tau (a). $$

Here, *λ*_*B*_ is the average underlying baseline *FOI* per person, *ζ*_*P*_ is the relative to baseline ratio of the average *FOI* after each round of treatment, with subscript *P* indicating the number of rounds of praziquantel treatments received, and the function *τ*(a) describes the (dimensionless) age-specific contact function normalized over the total host population. In order to use this continuous function of age in the modelling process, the human population is partitioned into *n* age groups each of width 1 year and mid-value *a*_1_ through *a*_*n*_ so that *n* = 60 and the upper end of the of the n-th class is 60 years, with *a*_*i*_ denoting the *i*-th age group,

3 $$ \tau \left({a}_i\right)=\frac{\rho \left({a}_i\right)}{{\displaystyle \sum_{a_i=1}^{a_i=n}\rho \left({a}_i\right)}}. $$

The function *ρ*(*a*_*i*_) represents the relative contact rates distributed over age, and following Chan et al. (1995) [39] it has equation,

4 $$ \rho \left({a}_i\right)={a}_i\;{e}^{-{\left(\beta {a}_i\right)}^2}+c. $$

The expression for *ρ*(*a*_*i*_) depends on two shape parameters, namely β and *c*, which together determine its functional form with host age. (Further details can be found in Supplementary Protocol S2 of [21].) The function *Λ*(*a*) denotes the yearly average number of (egg-producing) worms acquired per person of age *a* and comprises the product of the contact rate with infective stages, the probability of infection upon contact, and the average population of cercariae in the environment; information relating to the number of contacts per unit time is subsumed within *λ*_*B*_. We are not focussing on estimating absolute values of *FOI* but the relative changes at each point of follow-up. It is assumed that praziquantel instantaneously reduces the adult worm burden of *S. mansoni* by 95 % in all treated hosts [40].

At baseline (prior to treatment), *ζ*_0_ = 1, and at follow-up years 1, 2, and 3 (FY1, FY2 and FY3), *ζ*_1_, *ζ*_2_, and *ζ*_3_ indicate the ratio of the *FOI* relative to that at baseline, respectively, when data for all three follow-up years were available (e.g. Mali, Uganda). When follow-up data were available for one or two consecutive years after baseline (e.g. Zambia), the changes in the *FOI* were estimated as described for these years. For Tanzania, treatment was distributed at baseline and at FY1, but not at FY2 or FY3; however, evaluation was conducted at FY3, so an average change in the *FOI* for FY2 and FY3 is reported here. A value of *ζ*_*P*_ lower or greater than 1 indicates, respectively, a reduction or an increase in the *FOI* from baseline, and a statistically significant reduction or increase is indicated when the entire range of the confidence interval lies, respectively, belowor above 1. Confidence intervals for these parameters were estimated as outlined below.

**Fig. 2:**
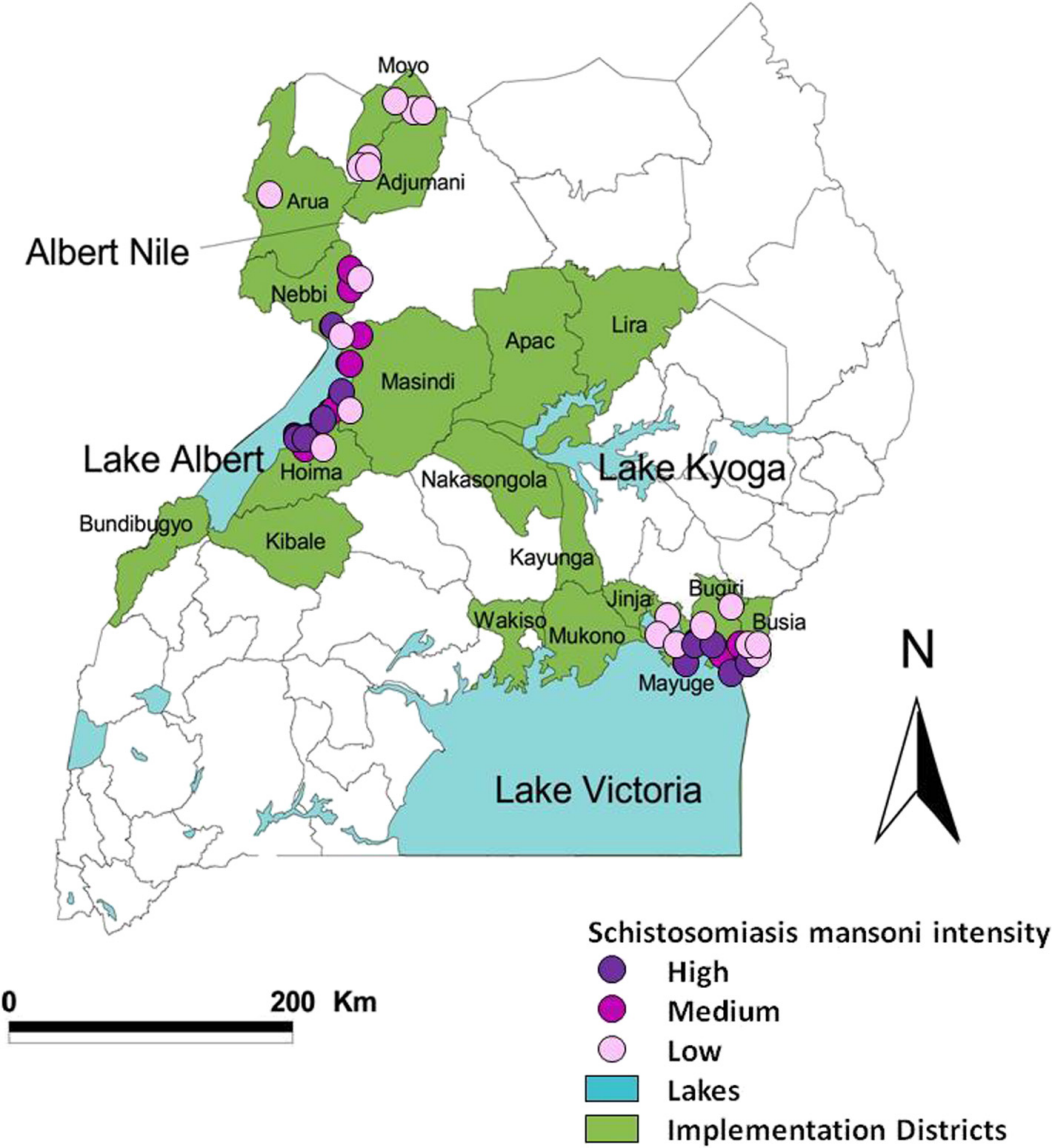
Map of Uganda showing results from baseline prevalence mapping of intestinal schistosomiasis in the country. The three main areas of schistosomiasis transmission are situated along the shores of Lake Victoria, Lake Albert, and Albert Nile. The three levels of *Schistosoma mansoni* endemicity at baseline are represented by closed circles: high (≥400 epg, violet); medium (100–399 epg, purple), and low (1–99 epg, pale pink) transmission. Figure reproduced with permission from Zhang et al*.* (2007)

To understand how the overall changes in the *FOI* at coarse-grain, macroepidemiological levels compared with those taking place at a fine-grain scale (which are more likely to correspond to individual transmission zones), changes in the *FOI* were also estimated separately for each of 32 Ugandan sentinel sites with longitudinal data and are reported where the sample size > 20 individuals (Fig. 2).

This dataset was also used to examine whether any programmatic variables were statistically associated with the *FOI*. A multivariate linear regression model incorporating random effects was used with baseline intensity of infection, treatment coverage and cohort retention rate at each time point as the covariates, and the absolute *FOI* at each sentinel site and time point as the dependent variable. Therapeutic treatment coverage (the proportion of people treated) was available only at the district level (not at the sentinel site level) and was calculated by dividing the number of people treated (taken from treatment registers) by the targeted number in each district (derived from census figures with a correction for population growth rates) (unpublished data). The random effects of district were included in order to control for any geographical clustering at this level [27]. Regression analyses were carried out at each of the three follow-up time points, and were weighted by the number of individuals at each sentinel site. The most parsimonious yet adequate model was chosen by comparing Akaike Information Criterion (AIC) scores [41]. *P*-values were considered significant at the 0.05 level.

#### *Schistosoma haematobium*

The model described in Eqs. 1, 2, 3 and 4 above was modified so the number of eggs (per 10 ml urine) produced per adult female worm was set to 3.60 [35], and the efficacy of praziquantel to 99 % [42]. This assumed efficacy was shown to provide a better fit to the data (see next subsection for a description of fitting approach) for the most complete *S. haematobium* dataset (that of Burkina Faso; log-likelihood ratio test statistic = 12.54, *p*-value of *χ*^2^ < 0.001) and was retained for all *S. haematobium* areas. This assumption is conservative with regards to estimates of reduction in the *FOI* following chemotherapy, i.e. the larger the assumed proportion of parasites killed with treatment, the greater the reinfection rate would need to be for parasite load to return to observed levels. *Schistosoma haematobium*-parameterised models were fitted to data from Burkina Faso and Tanzania (three consecutive years of follow-up), Mali and Niger (two years), and Zambia (one year).

### Fitting Approach

The cross-sectional cohorts at baseline consisted of both children and adults in order to provide the profile of age-related infection intensity for each schistosome species, endemicity area, and country. The age-stratified model of Eqs. 1, 2, 3 and 4 was fitted simultaneously to the longitudinal cohorts and the cross-sectional baseline data using maximum likelihood estimation of the parameters of interest. The model was fitted to individual host data (taking into account the high degree of parasite overdispersion observed and estimated in each dataset (see below and SI)) to estimate the following: the baseline *FOI*, *λ*_*B*_, the two shape parameters of the contact function (ββ and *c*– Eq. 4), and the change in the annual *FOI* after each round of chemotherapy (*ζ*_1_, *ζ*_2_, and *ζ*_3_) relative to that at baseline. Uncertainty around the parameters was estimated using the Fisher Information Matrix [43].

The multi-dimensional parameter space was explored using the Latin Hypercube sampling method [44, 45]. As part of this fitting approach, the infection intensity observations of individuals were compared to the model-derived, age-specific mean intensity of infection. Ninety five percent confidence intervals around each of the parameters were calculated using the Fisher Information Matrix [43]. Confidence intervals around the model output (mean egg output) were estimated by re-running the model and randomly selecting parameters from within their 95 % CI bounds. Runs which generated likelihood values not statistically significantly different from the best fit run (tested using a *Χ*^2^ distribution with the appropriate degrees of freedom) were used to construct 95 % CI around the model outputs [46]. The maximum and minimum mean egg output at each timepoint from these runs constituted the upper and lower confidence intervals respectively.

The *FOI* is a dynamic entity, decreasing immediately after treatment and increasing in inter-treatment periods. Therefore, the estimated values correspond to an average across the relevant periods (each period being one year for annual treatment unless otherwise stated) and were corrected for the ageing of the cohort. Given that these are longitudinal cohorts (with no replacement of the youngest ages), the average age of participants will inevitably increase, and schistosomiasis infection intensity is known to be strongly dependent on host age [17] with an increase in exposure typically experienced by children between the ages of 5 – 15 years. Therefore, the fitted mathematical model takes this into account by allowing the age of the cohort to increase over time. Not correcting for age can lead to underestimates of the *FOI* reductions (or overestimates if the *FOI* increases) [21].

Model-derived changes in the average infection intensity were calculated to help understand the impact of treatment. Of particular importance are the estimated changes in the proportion of individuals harbouring high infection intensity (defined as the prevalence of hosts excreting ≥400 epg for schistosomiasis mansoni and ≥50 e/10 ml for schistosomiasis haematobium), as those individuals are deemed to be the ones more likely to develop and suffer morbidity [47], though even light infections can be associated with significant morbidity [48].

Models were parameterized using parasite overdispersion values calculated from prevalence-intensity relationships for each endemicity area, parasite species, and country (data not shown). Overdispersion of schistosome distribution among hosts was assumed to be reasonably approximated by the negative binomial distribution, with the value of the overdispersion parameter (denoted by *k*, an inverse measure of the strength of overdispersion)*,* allowed to vary with the mean intensity of infection following either a linear (*k* = *k*_0_ + *k*_1_*m*), or a power function (*k* = *k*_0_ + *k*_1_*m*^*k*2^), where *m* denotes the arithmetic mean intensity of infection [49], measured in epg or e/10 ml.

### Ethical Approval

Data used for the analysis in this paper were collected as part of the routine monitoring and evaluation activities of the countries’ schistosomiasis control programmes. Ethical approval for this was provided by Imperial College Research Ethics Committee (ICREC_8_2_2, EC No. 03.36, R&D No. 03/SB/003E) and by the ethical review boards of the Ministries of Health of the respective endemic countries.

## Results

### Cohort representativeness

**Fig. 3:**
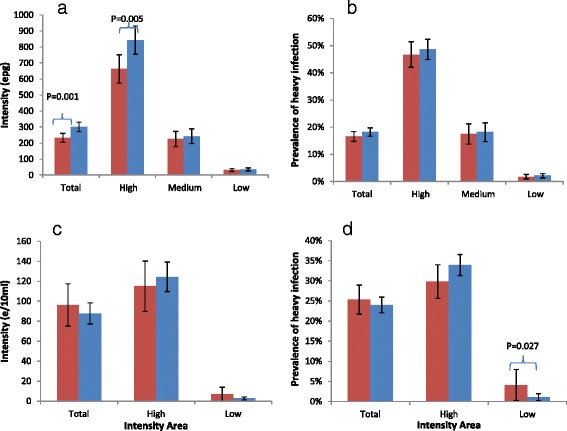
Differences in infection markers between those individuals followed up in the longitudinal cohort for all years (red bars), and those lost to follow-up (blue bars): **a** Mean infection intensity of *Schistosoma mansoni* in Uganda at baseline; **b** Prevalence of heavy intensity (≥400 epg) of *S. mansoni* in Uganda at baseline; **c** Mean infection intensity of *S. haematobium* in Burkina Faso at baseline; **d** Prevalence of heavy intensity (≥50 eggs/10 ml urine) of *S. haematobium* in Burkina Faso at baseline. *P-*Values are stated where differences are statistically significant (*P* ≤ 0.05), otherwise they are omitted

A comparison between the baseline values of infection intensity and prevalence of heavy infection in those individuals in the longitudinal cohort and those lost to follow-up was conducted using the most extensive datasets, namely the cohorts for *S. mansoni* in Uganda and for *S. haematobium* in Burkina Faso (Fig. 3). In Uganda (Fig. 3a and b) the intensity of infection was 29 % higher in those children who were lost to follow-up (301 epg [95 % CIs: 271, 330 egg] vs. 233 epg [95 % CIs: 205, 261], Z = 3.27, *p*-value = 0.001), driven primarily by a significant difference in the egg counts of high intensity infections (27.2 % higher; 843 epg [95 % CIs: 755, 930 egg] vs.663 epg [95 % CIs: 574, 752 egg], Z = 2.82, *p*-value = 0.005). However, there were no significant differences in the prevalence of heavy infection between the two groups (10.1 % higher in those lost to follow-up; 18.2 % [95 % CIs: 16.7 %, 19.8 %] vs.16.6 % [95 % CIs: 14.8 %, 18.3 %], Z = 1.41, *p*-value = 0.16).

In Burkina Faso, no significant differences were observed in infection intensity between those in the longitudinal cohort, and those who were lost to follow-up. With regard to the prevalence of heavy infection, there was a non-significant higher prevalence in those lost to follow-up in high intensity areas (33.9 % vs. 29.8 %, Z = 1.61, *p*-value = 0.10), and a statistically significantly lower prevalence in low intensity areas (1.09 % vs. 4.08 %, Z = 2.21, *p*-value = 0.02).

There was no significant difference in the cohort retention rate with respect to age in Uganda (logistic regression, coefficient = −0.0085, Z = −0.506, *p*-value = 0.61). However, in Burkina Faso the retention rate was statistically significantly and negatively associated with age (coefficient = −0.1165, Z = −2.888, *p*-value = 0.004).

### Changes in relative *FOI* with treatment

The relative changes in the *FOI* following successive treatment rounds for each country, schistosome species, and baseline endemicity areas are shown in Tables 2 and 3.

**Table 2:** Relative (to baseline) changes in the force of infection *(FOI)* and their 95 % confidence intervals estimated at the point of monitoring (one or two years) after each round of preventive chemotherapy with praziquantel for *Schistosoma mansoni* areas

Country	BL Infection Intensity Category	Number in cohort (n)	BL infection Intensity (epg)	BL *FOI* (wppy)	FY1 ζ_1_	FY2 ζ_2_	FY3 ζ_3_
Mali	High/Medium	215	452.75	16.35	0.97 [0.45, 1.92]	0.60 [0.41, 0.97]^a^	NA
Mali	Low	937	23.55	5.45	0.68 [0.45, 0.92]^a^	0.18 [0.09, 0.41]^a^	NA
Tanzania	High/Medium	290	400.38	18.26	3.61 [1.23, 5.04]^b^	2.03 [0.75, 3.22]	
Tanzania	Low	769	11.06	0.44	2.41 [1.23, 3.77]^b^	0.35 [0.15,0.78]^a^	
Uganda	High	262	766.04	30.00	0.78 [0.46, 1.50]	0.37 [0.22, 0.63]^a^	0.37 [0.19, 0.86]^a^
Uganda	Medium	235	231.69	10.02	1.04 [0.49, 2.00]	0.34 [0.13, 0.74]^a^	0.46 [0.17, 1.06]
Uganda	Low	600	33.54	1.69	0.24 [0, 0.61]^a^	0.32 [0.06, 0.64]^a^	0.20 [0.04, 0.43]^a^
Zambia	Medium	83	326.8	14.80	1.28 [0.52, 3.43]	NA	NA
Zambia	Low	1362	9.13	0.35	1.26 [0.89, 2.01]	NA	NA

*FOI*: force of infection expressed as the mean number of adult female parasites acquired per person per year; epg: eggs per gram of faeces; wppy: worm pairs per person per year; BL: baseline; FY1: follow-up year 1; FY2: follow-up year 2; FY3: follow-up year 3; ζ_1_, ζ_2_, ζ_3_: the relative change in the *FOI* at FY1, FY2, FY3 respectively, expressed as a proportion of that at baseline; ^a^significant decreases in *FOI* from baseline; ^b^significant increases in *FOI* from baseline: NA: not available. In Tanzania there was no treatment or survey at FY2 so the data collected at FY3 comprise an average across the previous two years

**Table 3:** Relative (to baseline) changes in the force of infection *(FOI)* and their 95 % confidence intervals estimated at the point of monitoring (one or two years) after each round of preventive chemotherapy with praziquantel for *Schistosoma haematobium* areas

Country	BL Infection Intensity Category	Number in cohort (n)	BL infection Intensity (epg)	BL *FOI* (wppy)	FY1 ζ_1_	FY2 ζ_2_	FY3 ζ_3_
					[95 % CI]	[95 % CI]	[95 % CI]
Burkina Faso	High	376	124.46	20.03	0.013 [0.0005, 0.12]^a^	0.124 [0.04, 0.21]^a^	0.048 [0.02, 0.12]^a^
Burkina Faso	Low	188	17.11	1.69	0.005 [0.0003, 0.14]^a^	0.253 [0.08, 0.56]^a^	0.001 [0.0003, 0.10]^a^
Mali	High	591	174.15	9.37	0.27 [0.11, 0.62]^a^	0.30 [0.11, 0.68]^a^	NA
Mali	Low	561	19.67	1.53	0.70 [0.32, 1.34]	0.17 [0.05, 0.44]^a^	NA
Niger	High	270	121.87	1.91	0.70 [0.23, 1.42]	0.86 [0.23, 1.88]	NA
Niger	Low	1047	20.76	0.63	0.18 [0.10, 0.35]^a^	0.36 [0.21, 0.76]^a^	NA
Tanzania	All (Low)	664	11.06	1.22	0.584 [0.22, 0.91]^a^	0.434 [0.21, 0.80]^a^	
Zambia	High	276	89.11	1.53	0.730 [0.21, 1.65]	NA	NA
Zambia	Low	1169	10.01	0.38	2.129 [0.89, 4.21]	NA	NA

*FOI* :force of infection expressed as the mean number of adult female parasites acquired per person per year; e/10 ml: eggs per 10 ml of urine; wppy: worms per person per year; BL: baseline; FY1: follow-up year 1; FY2: follow-up year 2; FY3: follow-up year 3; ζ_1_, ζ_2_, ζ_3_: the relative change in the *FOI* at FY1, FY2, FY3 respectively, expressed as a proportion of that at baseline; ^a^significant decreases in *FOI* from baseline: NA: not available. In Tanzania there was no treatment or survey at FY2 so the data collected at FY3 comprise an average across the previous two years. In Burkina Faso there was no treatment following FY1 so the results at FY1 and FY2 constitute two successive years following a single treatment round

### *Schistosoma mansoni*

Reductions in the *FOI* of *S. mansoni* in Uganda at a macroepidemiological level have been estimated previously [21]. In summary, large and statistically significant reductions were observed across all three infection intensity areas –following one round in low intensity areas (76 % reduction), and following two rounds in moderate (66 % reduction) and high (63 % reduction) intensity areas (Table 2). A similar situation was observed in Mali, with substantial and significant reductions following two rounds of treatment in low (82 % reduction) and high/moderate intensity areas (40 %). This contrasts with Tanzania where large increases in the *FOI* were observed following one round of treatment (although a second round resulted in sizeable reductions in low intensity areas). Modest increases in the *FOI* were observed in Zambia, both in the moderate and low intensity areas.

### *Schistosoma haematobium*

In Burkina Faso, very extensive reductions in the *FOI* were observed for *S. haematobium*, even following just a single round of treatment (Table 3), with infection and transmission levels dropping to virtually zero. Here, there was no treatment at FY1 so the values of ζ_1_ (1.3 and 0.5 %) and *ζ*_2_ (12.4 and 25.3 %) represent the relative ratio of the *FOI* one and two years after a single treatment round. Even after a two-year treatment gap, reinfection levels were markedly suppressed (close to 100 % reduction in high and low intensity areas in the first year; 88 and 82 % respectively in the second year). In Mali there were marked and statistically significant reductions following one round of treatment in high intensity areas, and following two rounds in low intensity areas. In Niger reductions were modest (and not statistically significant) in high intensity areas, and considerable and statistically significant in low intensity areas. In Tanzania there was a 50 % reduction following one and two rounds of treatment, which increased to 65 % following three rounds. Zambia presents a more confusing picture; there was a relatively small reduction in high intensity areas (27 %) following one round of treatment, but a large increase in the *FOI* in low intensity areas (more than twice the baseline value).

### Heterogeneity in changes in the *FOI* at a finer geographical scale

Changes in the *FOI* for each of the 32 sentinel sites from Uganda are displayed in Table 4. Heterogeneity was observed particularly in high intensity areas, where the *FOI* following one treatment round—as a proportion of that at baseline—ranged from 0.179 (i.e. an 82.1 % reduction) to 1.993 (i.e. a nearly twice as large a value as that at baseline). This variation may be due to true heterogeneity between the sites or the impact of small statistical size in some areas.

**Table 4:** Changes in infection intensity and *FOI* for each of 32 sentinel sites with longitudinal data collected prior and one year after praziquantel treatment as part of the monitoring and evaluation component of the Ugandan schistosomiasis control programme

BL infection intensity Category	District	Sentinel Site	No. in cohort (n)	BL *FOI* (wppy)	Infection intensity with *S. mansoni* (epg)				Ratio of *FOI* in relation to BL		
					BL	FY1	FY2	FY3	ζ1	ζ2	ζ3
High	Bugiri	Buyondo	75	26.97	444.4	59.4^a^	16.7^a^	3.5^a^	0.179^a^	0.035^a^	0.015^a^
		Wakawaka	30	30.01	746.3	384.8	539.1	160.0	1.574^b^	1.698^b^	0.945
	Busia	Buloosi	72	24.78	598.1	99.7^a^	52.4^a^	24.0^a^	0.357^a^	0.220^a^	0.099^a^
	Hoima	Kibiro	57	54.87	1000.3	408.6^a^	153.7^a^	247.5^a^	1.038	0.454^a^	0.553^a^
		Runga	20	47.35	710.4	404.6	230.4^a^	110.4^a^	1.444	0.976	0.727
		Tonya	9	61.81	1096.0	228.0^a^	48.0^a^	48.0^a^	0.566	0.088^a^	0.001^a^
	Masindi	Walukubwa	20	62.28	895.2	314.2^a^	108.9^a^	974.8	0.591	0.266^a^	1.346
	Mayuge	Bugoto LV	45	24.51	616.5	123.1^a^	61.8^a^	50.4^a^	0.484^a^	0.235^a^	0.085^a^
		Bwondha	33	28.94	529.5	83.3^a^	133.8^a^	61.1^a^	0.558^a^	0.604^a^	0.263^a^
	Nebbi	Panyimur	67	31.72	597.6	327.9^a^	84.6^a^	49.1^a^	1.993^b^	0.121^a^	0.189^a^
Medium	Bugiri	Busiro	57	12.06	154.9	141.4	51.1^a^	108.5	1.891^b^	0.572^a^	1.312
	Busia	Bwaniha	70	20.38	386.1	58.7^a^	25.0^a^	42.7^a^	0.513^a^	0.187^a^	0.251^a^
	Hoima	Kasenyi	55	15.46	299.1	347.5	208.9	201.3	1.626^b^	0.969	1.264
	Masindi	Butiaba	31	14.32	300.4	98.3	1.5^a^	27.0^a^	0.880	0.009^a^	0.424^a^
		Kabolwa	23	14.59	452.9	43.8^a^	0.0^a^	0.0^a^	0.198^a^	0.001^a^	0.001^a^
		Wanseko	57	10.12	114.2	31.2	20.6^a^	0.0^a^	0.465^a^	0.286^a^	0.000^a^
	Nebbi	Kinju	52	15.21	104.4	52.9	16.9^a^	81.1	1.196	0.444^a^	1.267
		Pokwero	59	4.86	105.8	29.0^a^	1.7^a^	0.0^a^	0.929	0.119^a^	0.023^a^
Low	Bugiri	Kibimba	41	0.36	13.5	16.2	15.4	0.0^a^	3.584^b^	2.214^b^	0.000^a^
		Kibuye	46	3.58	41.7	8.7	16.2	15.7	0.844	1.325	1.817^b^
	Busia	Maduwa	77	5.46	68.1	7.0^a^	3.9^a^	0.4^a^	0.772^a^	0.116^a^	0.010^a^
		Majanji	64	1.66	27.8	2.2	1.5	0.0	0.064^a^	0.059^a^	0.000^a^
	Hoima	Kibanjwa	103	0.02	0.2	0.0	0.0	0.0	0.000	0.000	0.000
	Mayuge	Bukizbu	59	0.40	7.7	0.6^a^	0.4^a^	0.0^a^	0.114^a^	0.135^a^	0.000^a^
		Ikulwe	46	0.04	0.5	0.0	0.0	-	0.246	0.000	-
		Lwanika	61	5.96	74.5	7.4^a^	3.5^a^	44.3	0.292^a^	0.222^a^	1.648^b^
	Moyo	Aliba	39	1.38	62.2	2.1^a^	5.7^a^	2.7^a^	0.056^a^	0.244^a^	0.103^a^
		Dufile	69	1.70	60.5	0.7^a^	4.1^a^	0.0^a^	0.008^a^	0.151^a^	0.000^a^
		Etele	71	0.16	7.4	8.7	16.4	0.0^a^	2.882^b^	9.789^b^	0.000^a^
		Laropi	68	1.40	41.6	0.0	1.1	0.0	0.000^a^	0.052^a^	0.000^a^
		Obongi	50	0.35	34.1	1.5	0.5	0.0	0.324^a^	0.10002^a^	0.000^a^
	Nebbi	Pagwaya	54	1.66	49.8	12.0	2.7^a^	2.1^a^	0.430^a^	0.088^a^	0.087^a^

*BL* baseline, *FOI* force of infection expressed as the mean number of adult female parasites acquired per person per year; wppy: worms per person per year; epg: eggs per gram of faeces, *FY1* follow-up year 1, *FY2* follow-up year 2, *FY3* follow-up year 3; ζ_1_, ζ_2_, ζ_3_: the relative change in the *FOI* at FY1, FY2, FY3, respectively, as a proportion of that at baseline; ^a^statistically significant reduction in infection intensity or *FOI* from baseline; ^b^statistically significant increase in infection intensity or *FOI* from baseline

Note that the decision to allocate each sentinel site into high, medium, and low infection intensity categories was based on the infection intensity of the overall school/community at BL, rather than that of the longitudinal cohort selected to be followed up from that school/community (e.g. Kabolwa is classified in the medium category as the school-level intensity at BL was 339.4 epg; the cohort level was 452.9 epg). Any cohort with fewer than 20 individuals was excluded

Significant increases in the *FOI* were observed in some sentinel sites/schools following treatment, although as may be expected the number of such sites diminished with successive treatment rounds. In Table 4, changes in the *FOI* are reported together with changes in the intensity of infection; there were no signficant increases in infection intensity following treatment (although there were some non-signficant increases) and reductions in infection intensity tended to increase in magnitude with successive treatment rounds.

### Factors influencing changes in the *FOI*

Treatment coverage data were collated at the district level in Uganda and the reported figures were generally high each year. At baseline, the average therapeutic coverage in those districts within which the sentinel sites were located was 83 % (ranging from 63 to 93 %), at FY1 it was 90 % (87–97 %), at FY2 82 % (63–99 %, and at FY3 86 % (78–98 %).

Results of multivariate regression analyses examining which programme-relevant factors may influence the *FOI* are shown in Table 5. No significant associations between district-level treatment coverage and the *FOI* were observed, or between the cohort follow-up rate (the retention rate of individuals in the cohort) and the *FOI*. At all three time points there was a statistically significant and positive association between the baseline intensity of infection and the *FOI* (FY1: estimate = 0.047, t-value = 6.434, *p*-value < 0.001; FY2: estimate = 0.022, t-value = 4.487, *p*-value < 0.001; FY3: estimate = 0.022, t-value = 3.187, *p*-value = 0.004).

**Table 5:** Coefficients (and associated *p*-values) for multivariate linear regression examining the association of programme-relevant covariates with the force of infection (*FOI*, measured as the parasite establishment rate). The *FOI* is estimated following 1, 2, and 3 rounds of praziquantel treatment at follow-up years FY1, FY2, FY3 in Uganda

Covariates	Dependent Variable		
	FY1 *FOI*	FY2 *FOI*	FY3 *FOI*
Baseline Coverage	16.27 (0.756)	25.40 (0.355)	−26.77 (0.721)
Baseline Intensity	0.047 (<0.001)	0.022 (<0.001)	0.022 (0.043)
BL - FY1 Cohort Follow-up Rate	−9.502 (0.722)	−17.39 (0.282)	−5.609 (0.857)
FY1 Coverage	-	34.87 (0.473)	11.992 (0.882)
FY1 - FY2 Cohort Follow-up Rate	-	−0.318 (0.993)	−2.807 (0.961)
FY2 Coverage	-	-	30.972 (0.457)
FY2 - FY3 Cohort Follow-up Rate	-	-	−14.802 (0.287)

For model selection, the model that best described the data was selected for each timepoint separately, using a step function in R to compare AIC values. For the *FOI* at FY1, the best fitting model included only the baseline infection intensity of the cohort (estimate = 0.047, t-value = 6.66, *p*-value < 0.001). The same situation was found for the *FOI* at FY2 (estimate = 0.0218, t-value = 4.70, *p*-value < 0.001) and at FY3 (estimate = 0.0236, t-value = 3.74, *p*-value = 0.001).

### Changes in intensity of infection

**Fig. 4:**
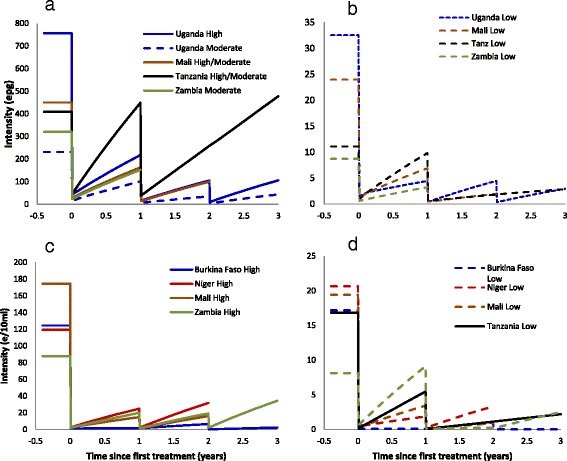
Changes in egg count for each of the countries and endemicity areas under praziquantel treatment in: **a** and **b** *Schistosoma mansoni* endemic areas (intensity measured as eggs per gram of faeces), and in **c** and **d** *S. haematobium* endemic areas (intensity measured as eggs per 10 ml urine). Data were collected annually. The lines linking the data points are included for illustrative purposes are not verified parasitologically. These lines assume a constant force of infection and assume 95 % efficacy of treatment for *S. mansoni* and 99 % efficacy of treatment for *S. haematobium*

The temporal trends of infection intensity following treatment are shown in Fig. 4. Upon treatment an instantaneous reduction in infection is modelled, followed by reinfection throughout the year prior to the next treatment round. For *S. mansoni* infections, significant overall reductions were observed in all countries except in Tanzania, where the intensity of infection remained stable throughout the monitored duration of control (for high/moderate intensity areas; Fig. 4a), or stable after one treatment and reduced after two treatment rounds (low intensity areas; Fig. 4b). Similarly, reductions in the infection intensity of *S. haematobium* were observed in all countries except in those areas of Zambia with a low intensity of infection at baseline, where there was a slight increase following a single round of treatment (Fig. 4d).

### Reduction in the prevalence of heavy infection

**Fig. 5:**
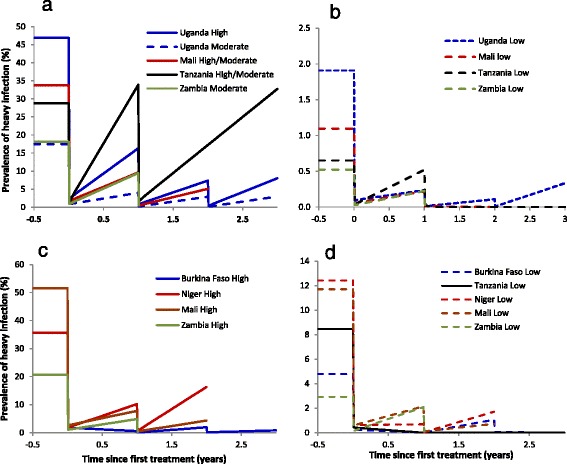
Changes in the model-derived estimate of prevalence of heavy infection (*S. mansoni* ≥400 epg; *S. haematobium* ≥50e/10 ml) with treatment with praziquantel. **a** and **b** *S. mansoni* areas; **c** and **d** *S. haematobium* areas. Note difference in y-axis scales. Data were collected annually. The lines linking the data points are included for illustrative purposes are not verified parasitologically. These lines assume a constant force of infection and assume 95 % efficacy of treatment for *S. mansoni* and 99 % efficacy of treatment for *S. haematobium*

As a proxy for morbidity, the proportion of individuals harbouring ‘heavy’ parasite burden (itself measured by egg load excreted in stool and urine) fell sharply in most areas (Fig. 5). For *S. mansoni* there were substantial reductions in Uganda and Mali, and slight increases in Tanzania, where it remained around 30 % in high/moderate intensity areas, and around 0.5 % in low intensity areas. For *S. haematobium*, the prevalence of heavy infection intensity fell everywhere, including in Zambia (where there had been relative increases in the *FOI*), from 20.5 to 4.8 % in high/moderate intensity areas and from 2.9 to 2.1 % in low intensity areas.

## Discussion

The impact of preventative chemotherapy with praziquantel on the level of schistosomiasis infection intensity, prevalence and morbidity in SCI-assisted programmes has been published previously (e.g. [2] and references therein). In this paper, we assessed the impact on transmission according to schistosome species, baseline endemicity level, country (and sentinel site for Uganda) for six SSA countries that have been assisted by the SCI to implement MDA programmes with praziquantel.

### Representativeness of the SCI longitudinal cohorts

In areas with higher infection intensity, children lost to follow-up had a significantly higher parasite load than those who remained in the cohort. These differences may have been caused by those heavily infected children being too ill to come back for treatment, being less likely to be enrolled in schools or having a higher degree of absenteeism. In Uganda, the higher infection intensity in those lost to follow-up could also have been caused by the movement of peripatetic fishing communities who historically suffer from high infection intensity and present only occasionally for treatment. Conversely, where infection levels were higher in the longitudinal cohort in areas of low endemicity, infected children may have been more likely to feel unwell and therefore more likely to seek treatment than their uninfected peers.

There was no difference in cohort retention rate with host age in Uganda. However, in Burkina Faso older children were less likely to return for treatment at each follow-up year. As older children typically have heavier infections, infection intensity in follow-up years may have been underestimated, leading to overestimations of the reductions in the *FOI*, although the differences in follow-up were small and the age-range relatively narrow.

It may be argued that those individuals recruited into the SCI cohorts do not represent the wider community because school-aged children are exposed to more extensive health messages around avoiding risky water contact. One approach to overcome this potential bias is to use putatively untreated 6-year olds recruited into the cohort each year as a proxy for the community. This approach was attempted in an earlier paper [21] but was not as useful as expected, likely due to the uncertainty about their treatment and infection history.

### Variation in relative changes of the *FOI* at broader- and fine-grain epidemiological levels

Reductions in the *FOI* relative to baseline values were observed in most, but not all, combinations of country, species, endemicity levels, and treatment rounds. Such reductions will benefit not only those who receive treatment, but also those individuals living in the same area (or accessing the same transmission site) who do not receive treatment, including non-enrolled school children who do not present for treatment, those too young to be included in MDA programmes, or adults living in an area receiving school-based treatment only. These benefits are often missed from the evaluation of schistosomiasis (and other helminthiases) control programmes (though see [50]). Exceptions to these reductions are reported for Tanzania (particularly for *S. mansoni* and *S. haematobium* to a lesser extent), and Zambia (for *S. mansoni* and *S. haematobium* in low intensity areas), where relative increases in the *FOI* were observed (although the average intensity of infection remained stable or dropped). The results from the control programme in Tanzania were affected by very low coverage rates in some areas (of the order of 10–20 %, unpublished data), due to adverse publicity against the aims of the control programme, thought to have been instigated as part of a politically-motivated campaign [51]. A further possible explanation is that of treatment failure in these areas. However, evidence to the contrary is provided by population genetic studies of schistosomes from Tanzanian schools [52], which demonstrated that parasites obtained from children after treatment were not closely related to those obtained at baseline, suggesting reinfection rather than clearance failure. Increases in the *FOI* were recorded in Zambia although the average infection intensity was reduced significantly in the cohorts followed up for *S. mansoni* and only increased marginally significantly in the *S. haematobium* cohort. In low intensity areas, treatment was targeted at school-aged children only, which will likely lead to a smaller impact on infection intensity/*FOI* as only a smaller proportion of the circulating pool of parasites would be cleared by praziquantel.

It cannot be discounted that the differences in sampling strategy (e.g. number of Kato Katz slides taken from a number of stool sample) had an impact on the estimates of parasite intensity and therefore on changes in the *FOI*. This will be the subject of a future analysis.

Considerable heterogeneity in relative changes in the *FOI* was also observed at the sentinel site/school level in Uganda. Possible explanations include variation in epidemiological, ecological, programmatic (e.g. levels of compliance) and locale-specific factors. In addition, variability will inevitably be introduced by the stochastic nature of the infection process given the smaller size of these cohorts. The extent of the heterogeneity between these sites provides a cautionary note to the interpretation of the results of control programmes at a more macroepidemiological level, i.e. schistosomiasis is fundamentally a focal disease. This is particularly important when examining the impact of control interventions at the level of transmission zones or contemplating the possibility of elimination, as the presence of transmission hot-spots and the possible connectivity between these through movement of definitive and/or intermediate hosts may help maintain and resupply the infection [53].

### Factors influencing changes in the *FOI*

The lack of clear relationships between the estimated *FOI* and programmatic factors such as treatment coverage was perhaps surprising, although this was undoubtedly affected by the relatively small range of treatment coverage observed, its aggregation level (district rather than school/community), and its dependence on dated census reports. Further work is required as and when treatment coverage data become available at a more disaggregated level. In other helminth diseases relying on MDA for their control (e.g. onchocerciasis), not only are the levels of therapeutic coverage important, but those of systematic non-compliance are crucial in influencing the long-term trends of infection intensity and prevalence as shown in modelling studies [54, 55]. However, the relationship between the *FOI* and the baseline endemicity level is interesting and consistent with findings in other helminth infections for which the duration of treatment necessary to eliminate the infection for various initial endemicity levels has been investigated [56].

### Model structure and parasite biology

Worm lifespan is a parameter that plays a key role in driving the outcomes of the model; the shorter the lifespan the higher the corresponding estimates of the *FOI*, yet there is still significant uncertainty as to the value for this parameter, with 2 to 10 years often quoted as a plausible range for the mean life span [3, 4]. However, some reported cases of infection (in the absence of reinfection) exceed 30 years [57, 58], suggesting that the distribution may be heavily right-skewed. Studies to elucidate the patterns of worm lifespan are required [59], not just in terms of average age, but also the shape of the distribution of worm ages. This will be important to consider for any future elimination programmes in order to predict the required length of control interventions (e.g. vector control, chemotherapy), as has been the case in other helminth infections such as lymphatic filariasis [60] and onchocerciasis [61, 62], particularly in view of the fact that schistosomiasis programmes are starting to consider elimination of the infection as a goal [63, 64].

There are likely to be density-dependent processes operating on the worm’s lifecycle, such as mating probability, parasite establishment (via acquired immunity elicited by established worms), and parasite fecundity. A reduction in parasite intensity following treatment will lead to a relaxation of the negative density-dependent processes (an increase in the per-capita establishment or reproductive success of the parasite), and therefore smaller reductions in *FOI* than might otherwise be expected. The modelling approach utilised here defines the *FOI* in terms of the number of worms which establish per host per unit time which encapsulates all of the above processes (as has also been utilised in onchocerciasis studies [65], rather than in terms of the host’s exposure to parasite transmission stages, which is sometimes referred to as the transmission potential [66, 67]. We contend that this definition is more useful for morbidity control programmes as it is the established worms that drive morbidity and onward transmission, rather than the parasites to which hosts are exposed.

We still have an incomplete understanding of how the immunity of human hosts influences the rate of reinfection following treatment. Therefore, changes in *FOI* need to be interpreted carefully. The reduced rate of reinfection following treatment could be explained either through a decrease in environmental transmission or a decrease in the treated host population’s susceptibility to reinfection. PZQ treatment is known to release somatic parasite antigens which may elicit protective responses that facilitate resistance to reinfection. However, it is far from clear how much this immunological response may influence the rate of *Schistosoma* establishment, development, or fecundity.

### Parasite ‘strains’ and hybridization

There is an increasing body of evidence for the existence of some degree of within-species differentiation in the *Schistosoma* genus. Differing phenotypes and genotypes of parasites have been reported as occurring in different regions of SSA (for *S. mansoni* [68, 69], for *S. haematobium* [69–71] and for *S. japonicum* [72]). It is likely that such parasite ‘strains’ may vary in their susceptibility to praziquantel. Epidemiological and ecological conditions, such as water-contact behaviour, the resilience of snail intermediate hosts to environmental perturbations, and the extent and permanence of water bodies will inevitably differ between areas. In addition, secular changes such as rainfall patterns; water and sanitation programmes; human population distribution and migration; patterns of coinfection; and the motivation and experience of the programme staff will all affect the success of a control programme. These all constitute examples of the challenges in translating what are undoubtedly clinically efficacious interventions into effective community-level programmes [73].

Allayed to this is the issue of hybridization between schistosome parasite species, often between human and non-human schistosome species. There is reported evidence of natural hybridization between *S. haematobium* and the livestock species *S. bovis* in Senegal [74], between *S. haematobium* and *S. guineensis*in Cameroon [75, 76], between *S. haematobium* and *S. curassoni* of livestock in Senegal [77], between *S. mansoni* and the rodent parasite *S. rodhaini* in Kenya [78], and of mating interactions between *S. mansoni* and *S. haematobium* in hamster models [79]. Additionally, co-infection of *S. mansoni* and *S. haematobium* has been found to have an impact on the infectivity and morbidity of single versus mixed infections [80–82].

### Control versus elimination

Although annual praziquantel treatment resulted, in the majority of areas, in relative reductions in the *FOI*, in the absence of on-going treatment and/or measures that more permanently reduce exposure, parasite acquisition and infection intensity will likely return towards baseline values. Recently there has been an increased focus on elimination of schistosomiasis using a combination of intervention strategies, such as has occurred in Morocco [83, 84], Japan [85], and in large areas of China [86]. How feasible elimination of infection is in the transmission heartland of SSA is less certain. However, there are now programmes that aim to identify the kinds of integrated strategies (such as MDA, water and sanitation improvement, and comprehensive health education messages) that are needed to eliminate schistosomiasis in SSA, particularly in more isolated foci such as on the Unguja island of Zanzibar [63, 64].

The results from the models presented here can help towards this end by identifying areas where MDA alone may be sufficient to push transmission below thresholds of no return (known as transmission breakpoints [10, 11]), or more pragmatically, below operational thresholds for elimination that would indicate the cessation of MDA and the commencement of post-MDA surveillance. This approach has been taken in other helminthiases such as onchocerciasis to identify, through modelling studies, epidemiological scenarios that would require annual or biannual MDA, or the addition of other complementary interventions [55, 87]. We advocate that further modelling work be conducted, including the development of stochastic frameworks, to examine the effect of multiple interventions and of multiple hosts [88] that could be deployed concurrently or in staggered regimes, and to identify transmission breakpoints in each area, as well as the impact of chance events such as stochastic fade-out.

## Conclusions

Significant reductions in the *FOI* compared to baseline values, as estimated using a schistosomiasis transmission model, were reported in many, but not all, of the SCI cohorts across SSA for both *S. mansoni* and *S. haematobium* infections following successive rounds of praziquantel treatment. These reductions will benefit those individuals who receive treatment as well as those who do not, an aspect of MDA that is often missed from its analysis and advocacy, and which is crucial for the quantification of the cost effectiveness of interventions [89]. A wide range of values were observed, likely reflecting the locale-specific ecological, epidemiological, and programmatic conditions, such as the differing approaches to implementation and successes of the various countries’ control programmes. Changes in the *FOI* at a finer scale also showed great heterogeneity, reflecting the focality of schistosomiasis transmission, and the stochastic nature of the infection process. The application of transmission dynamics models, fitted to longitudinal M&E data, constitutes a helpful tool in the evaluation of large-scale schistosomiasis control and elimination programmes.
